# Gpnmb Is a Potential Marker for the Visceral Pathology in Niemann-Pick Type C Disease

**DOI:** 10.1371/journal.pone.0147208

**Published:** 2016-01-15

**Authors:** André R. A. Marques, Tanit L. Gabriel, Jan Aten, Cindy P. A. A. van Roomen, Roelof Ottenhoff, Nike Claessen, Pilar Alfonso, Pilar Irún, Pilar Giraldo, Johannes M. F. G. Aerts, Marco van Eijk

**Affiliations:** 1 Department of Medical Biochemistry, Academic Medical Center, 1105 AZ, Amsterdam, The Netherlands; 2 Department of Pathology, Academic Medical Center, 1105 AZ, Amsterdam, The Netherlands; 3 Centro de Investigación Biomédica en Red de Enfermedades Raras, Unidad de Investigación Traslacional, Zaragoza, Spain; 4 Department of Biochemistry, Leiden Institute of Chemistry, Leiden University, 2300 RA, Leiden, The Netherlands; University Hospital S. Maria della Misericordia, Udine, ITALY

## Abstract

Impaired function of NPC1 or NPC2 lysosomal proteins leads to the intracellular accumulation of unesterified cholesterol, the primary defect underlying Niemann-Pick type C (NPC) disease. In addition, glycosphingolipids (GSLs) accumulate in lysosomes as well. Intralysosomal lipid accumulation triggers the activation of a set of genes, including potential biomarkers. Transcript levels of Gpnmb have been shown to be elevated in various tissues of an NPC mouse model. We speculated that Gpnmb could serve as a marker for visceral lipid accumulation in NPC disease. We report that Gpnmb expression is increased at protein level in macrophages in the viscera of *Npc1*^*nih/nih*^ mice. Interestingly, soluble Gpnmb was also found to be increased in murine and NPC patient plasma. Exposure of RAW264.7 macrophages to the NPC-phenotype-inducing drug U18666A also upregulated Gpnmb expression. Inhibition of GSL synthesis with the glucosylceramide synthase (GCS) inhibitor N-butyl-1-deoxynojirimycin prevented U18666A-induced Gpnmb induction and secretion. In summary, we show that Gpnmb is upregulated in NPC mice and patients, most likely due to GSL accumulation.

## Introduction

Niemann-Pick type C (NPC) disease is an autosomal recessive neurovisceral lysosomal storage disorder (LSD) with an estimated minimal incidence of 1/120 000 live births [1]. Loss-of-function mutations in the transmembrane protein NPC1 account for approximately 95% of the NPC cases, with the remainder involving the soluble protein NPC2 [2]. NPC1 and NPC2 are thought to function cooperatively in the transport of cholesterol from late endosomal/lysosomal (LE/L) compartment to other cellular sites [3]. Malfunction of either protein leads to the accumulation of unesterified cholesterol in LE/L. Glycosphingolipid (GSL) accumulation also occurs, most likely as a result of impaired activity of multiple lysosomal hydrolases [4–6].

The disease manifestation is heterogeneous with respect to phenotype, progression and age of onset. Four different forms can be classified based on the onset and severity: the early-infantile form, in which patients usually die before the age of 5; the late-infantile form with severe liver and pulmonary affection; the juvenile or classic form, in which motor skill and neurodegeneration disorders manifest in middle to late childhood patients; the adult form, in which patients present neurologic disease and progressive dementia [1]. The only therapy for NPC approved by the European Medical Association (EMA) is oral administration of the glucosylceramide synthase (GCS) inhibitor N-butyl-1-deoxynojirimycin (NB-DNJ, Zavesca) [7]. NB-DNJ inhibits the synthesis of glucosylceramide (GlcCer), the building-block of more complex GSLs, but does not directly target the unesterified cholesterol accumulation.

The spontaneously occurring BALB/cNctr-*Npc1*^*m1N*^/J (*Npc1*^*nih/nih*^) mouse strain is defined by an early truncation of the NPC1 protein [8]. This strain displays all the hallmarks of the human infantile form of NPC, including increased hepatic fibrosis and oxidative stress markers, as well as progressive hepatomegaly due to the accumulation of free cholesterol [9,10]. The *Npc1*^*nih/nih*^ mice also demonstrate alterations in immune cell phenotypes in the brain, liver and spleen [10–14]. For instance, macrophage foam cells are present in the liver [15] and natural killer T cell development is impaired [16]. Recently it has been demonstrated that inflammation occurs secondarily to neuronal and epithelial cell dysfunction in NPC mice [11].

Two diagnostic standards for NPC1 are currently used. The first one requires a skin biopsy and subsequent demonstration of lysosomal cholesterol accumulation in cultured fibroblasts by filipin staining. The second procedure is based on demonstration of elevated plasma oxysterol levels, requiring advanced quantitative mass spectrometry [17]. The lack of more convenient blood based diagnostic markers for NPC triggered various genomic and lipid-based approaches searching for new serum biomarkers in NPC mouse models [18–20]. Recently, the lipid lyso-sphingomyelin and its isoform lyso-sphingomyelin-509 have been shown to have great potential as diagnostic biomarker for NPC [21,22]. Genomic studies yielded various candidates, including cathepsins, lysozyme and galectin-3 [18–20,23,24]. Glycoprotein nonmetastatic melanoma protein B (Gpnmb) mRNA was also found to be increased in *Npc1*^*nih/nih*^ mouse brain, liver and spleen [18].

Although Gpnmb is a membrane protein, it has been observed that cell types such as C2C12 myoblasts, melanocytic cells, astrocytes and breast cancer cells release by shedding a soluble form of Gpnmb [25–28]. Here we investigated the potential of Gpnmb as a plasma marker to follow disease progression in mouse models or previously diagnosed cases of NPC.

## Materials and Methods

### Cell culture

RAW264.7 cells were obtained from the American Type Culture Collection and were cultured in DMEM (Dulbecco’s Modified Eagle Medium)/10% FCS (fetal calf serum) supplemented with penicillin/streptomycin. *In vitro* the NPC phenotype was induced with U18666A (Sigma) at concentrations ranging from 1–10 μM; N-butyl-1-deoxynojirimycin (Sigma) was used at 250 μM to block GSL synthesis.

### Animals

*Npc1*^*nih/nih*^ and mice *Npc1*^nmf164^, along with wild-type littermates (*Npc1*^+/+^), were generated by crossing *Npc1*^+/nih^ or *Npc1*^+/nmf164^ males and females in-house. The heterozygous BALB/c Nctr-*Npc1*^*m1N*^/J mice (stock number 003092) and C57BL/6J-*Npc1*^*nmf164*^/J (stock number 004817) were obtained from The Jackson Laboratory (Bar Harbor, USA). Mouse pups were genotyped according to published protocols [8,29]. Mice (± 3 weeks old) received the rodent AM-II diet (Arie Blok Diervoeders, Woerden, The Netherlands). The mice were housed at the Institute Animal Core Facility in a temperature- and humidity-controlled room with a 12-h light/dark cycle and given free access to food and water ad libitum. All animal protocols were approved by the Institutional Animal Welfare Committee of the Academic Medical Centre Amsterdam in the Netherlands (DBC101698 and DBC17AC). Animals were first anesthetized with a dose of Hypnorm (0.315 mg/mL phenyl citrate and 10 mg/mL fluanisone) and Dormicum (5 mg/mL midazolam) according to their weight. The given dose was 80 μL/10 g bodyweight. Animals were sacrificed by cervical dislocation. Organs were collected by surgery and rinsed with PBS. Each one was sliced in half: one part was directly snap-frozen in liquid nitrogen and stored at -80°C for lipid and gene expression analysis and the other half was conserved in phosphate-buffered formalin for posterior histologic analysis. Later, homogenates for lipid analysis were made from the frozen material in 25 mM potassium phosphate buffer, pH 6.5, supplemented with 0.1% (v/v) Triton X-100 and protease inhibitors.

### Patients

EDTA plasma samples of 17 NPC patients and 8 NPC carriers were collected prior to therapy initiation at the Unidad de Investigación Traslacional in Zaragoza. The status of affected or carrier of NPC disease was determined after the exomic sequencing of NPC1 and NPC2 genes, according to the presence of two or one mutations, respectively. Filipin stainings of fibroblasts were conducted to complete the diagnosis study. Plasma samples were stored frozen at -20°C until further use. Plasma samples of 5 male and 4 female control subjects were collected at the Academic Medical Center. Plasma samples were stored frozen at -20°C until further use.

### Ethics statement

A written informed consent was obtained from each patient or their guardian. The study protocol was approved by the Ethics Committee for Clinical Investigation of Aragon (CEICA) in Spain, and the study was carried out in accordance with the ethical standards of the Declaration of Helsinki. A written informed consent was also obtained from each healthy volunteer. The study was approved by the Medical Ethics Committee of the Academic Medical Center in Amsterdam, the Netherlands, and was conducted according to the Declaration of Helsinki principles.

### Immunohistochemistry

For histology, 4-μm-thick sections were cut from formalin-fixed, paraffin-embedded tissue and mounted on glass slides. After deparaffinization and rehydration, sections were stained with hematoxylin and eosin (HE). For immunohistology, heat-induced epitope retrieval (HIER) of rehydrated sections was performed, using 10 mM citrate, pH 6.0, for 10 min at 98°C. After washing, the sections were subsequently incubated with antigen affinity-purified polyclonal goat IgG anti-Gpnmb (AF2330; R&D Systems, Abingdon, UK), washed, incubated with rabbit anti-goat IgG (6160–01; Southern Biotech, Birmingham, AL, USA), washed, and incubated with intestinal alkaline phosphatase-conjugated poly-AP goat anti-rabbit IgG (BrightVision; ImmunoLogic, Klinipath, Duiven, The Netherlands). Intestinal AP activity was detected using VectorBlue substrate (SK-5300; Vector Laboratories, Burlingame, CA, USA) in presence of 0.2 mM levamisole to inhibit endogenous non-intestinal alkaline phosphatase activity. After washing, a second HIER was applied to denature and remove all antibodies from the first staining sequence while leaving the precipitated chromogen unchanged [30]. Subsequently, sections were incubated with rabbit anti-ionized calcium binding adapter molecule 1 (Iba1) (#019–19741; WAKO Chemicals, Neuss, Germany), washed, and incubated with poly-AP goat anti-rabbit IgG. AP activity was detected using VectorRed substrate (SK-5100; Vector Laboratories) in presence of 0.2 mM levamisole. Sections were counterstained with methyl green and mounted with VectaMount (Vector Laboratories). Analysis was performed using brightfield microscopy (Leica DM5000B) with an HC PLAN APO 20x/0.70 objective. Multispectral data sets were acquired using a Nuance imaging system (Perkin Elmer, Hopkinton, MA, USA) from 420 to 720 nm at intervals of 10 nm. Spectral libraries for each chromogen were obtained from single-stained sections and were used to unmix the triple staining patterns. Nuance 3.0.2 software was used to display intensity heat maps for single channels and to construct composite images applying colour universal design.

### Measurement of Neutral (Glyco)sphingolipids

Ceramide (Cer), glucosylceramide (GlcCer), lactosylceramide (LacCer) and globotriaosylceramide (Gb3) levels were determined as described previously [31]. Shortly, lipids were extracted according to Bligh/Dyer et al [32], Chloroform phase was thoroughly dried, and deacylation of lipids was performed in 0.5 mL of 0.1 M NaOH in methanol in a microwave oven (CEM microwave Solids/Moisture System SAM-155). Deacylated lipids were derivatized on-line for 30 min with O-phtaldehyde and separated with a HPLC system (Waters Associates, Milford, USA) and a Hypersil BDS C_18_ 3 μm, 150 mm x 4.6 mm reverse-phase column (Alltech Inc., USA). All samples were measured in duplicate (tissues samples) or triplicate (cellular lysates), and in every run a reference sample was included. Sphingosine levels were measured as described above, but skipping the deacylation step.

### Cholesterol measurement

Total cholesterol levels in liver and spleen homogenates were extracted with chloroform/methanol according to Folch [33] followed by quantification using a colorimetric enzymatic kit (Biolabo, Maizy, France). Total cholesterol levels in cell lysates were determined using a modified enzymatic fluorometric assay previously described [34]. Briefly, cells were washed three times in PBS and lysed in water. Total cholesterol levels in the cell lysates were determined by incubating samples with 0.03U of cholesterol oxidase (*Streptomyces sp*., Sigma) and 0.005U of cholesterol esterase together with peroxidase, taurocholate and homovanillic acid in a 0.1M MOPS buffer (pH 7.7) containing 0.1% (v/v) Triton X-100. Formed fluorescence was measured in Synergy HT (Molecular Devices, BioTek Instruments, Winooski, VT, USA) multi-detection microplate reader with excitation at 320 nm and emission at 440 nm.

### Gene expression

Total RNA was extracted from liver and spleen combining the TRIZOL (Invitrogen) reagent and the nucleospin II extraction kit (Macherey-Nagel) as described previously [35]. RNA concentrations were measured using the Nanodrop Spectrophotometer (Nanodrop Technologies). Equal amounts of RNA were used to synthesize cDNA, according to the manufacturer’s method (Invitrogen). Gene specific analysis was done by real-time RT-PCR using an iCycler MyiQTM system (Biorad). Expression levels of Gpnmb (forward 5’-caggaatgatttgggactgacc-3’; reverse 5’-ccgggaacctgagatgctg-3’), Ccl3 (forward 5’-ccaagtcttctcagcgccat-3’ reverse 5’-tcttccggctgtaggagaagc-3’), CtsD (forward 5’-gctccttctccactgtcagg-3’; reverse 5’-ctggctggcttcctctactg-3’), Iba1 (forward 5’-ggatttgcagggaggaaaag-3’; reverse 5’-tgggatcatcgaggaattg-3’) were normalized to those of acidic ribosomal phosphoprotein 36B4 also referred to as P0 (forward 5’-ggacccgagaagacctcctt-3’ reverse 5’-gcacatcactcagaatttcaatgg-3’).

### Protein concentration

Determined using the Pierce BCA Protein Assay kit (Thermo Scientific^®^) by the microplate procedure. Absorbance measured in EL808 Ultra Microplate Reader (BIO-TEK Instruments Inc.) at 550nm.

### Western blotting

Total cell lysates were prepared in RIPA buffer (150 mM NaCl, 50 mM Tris—HCl pH 7.4, 2 mM EDTA, 0.5% deoxycholate, 1mM Na3VO4, 20 mM NaF, 0.5%Triton X-100), supplemented with protease inhibitor cocktail (Roche Applied Science) and phenylmethylsulfonyl fluoride (PMSF). Lysates were cleared by centrifugation at 4°C for 10 min at 10,000 × *g*. Equal amounts of protein were subjected to electrophoresis on 10% SDS-polyacrylamide gels and then transferred to nitrocellulose membranes (Whatman, Dassel, Germany) using an electroblotting apparatus (Bio-Rad Laboratories, Hercules, CA, USA). Primary antibodies used were the polyclonal anti-Gpnmb (R&D Systems) and anti-tubulin-alpha (Cedarlane Laboratories Limited). Matching secondary IRDye-conjugated antibodies (Westburg) were used for detection in an Odyssey V3.0 (LI-COR Inc.).

### ELISA Gpnmb (mouse and human)

Levels of soluble Gpnmb (sGpnmb) were determined using mouse or human specific ELISAs (R&D Systems).

### Chitotriosidase activity

Chitotriosidase was determined as previously described [36].

### Statistical Analysis

Values in figures are presented as a mean ± S.E.M. (cell experiments) or as a mean ± S.D. (animal experiments). Data were analysed by unpaired Student’s t-test or two-way ANOVA as indicated. Soluble Gpnmb was analysed by Mann-Whitney u-test. P values < 0.05 were considered significant. Pearson correlational analysis was used to evaluate correlation as indicated.

## Results

### *Npc1*^nih/nih^ mice progressively accumulate cholesterol and (glyco)sphingolipids in liver and spleen

**Fig 1:**
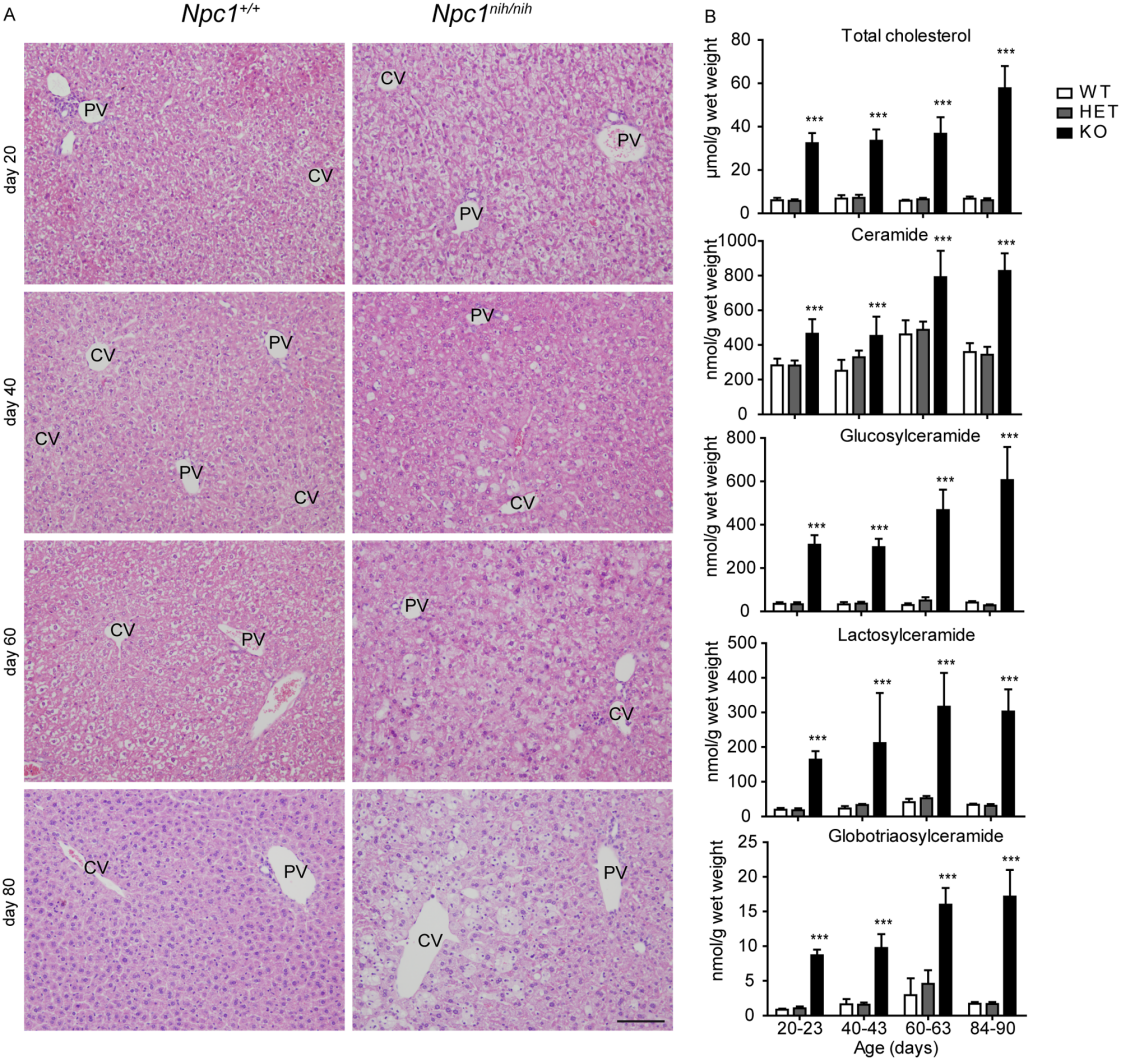
Progressive accumulation of cholesterol, GSLs and foam cells in *Npc1*^*nih/nih*^ mice liver. (A) H&E staining of liver sections of *Npc1*^+/+^ and *Npc1*^*nih/nih*^ at 20, 40, 60 and 80 days of age. CV = central vein, PV = portal vein. Scale bar = 100 μm. (B) Levels of total cholesterol, ceramide and GSLs, glucosylceramide, lactosylceramide and globotriaosylceramide in liver lysates of *Npc1*^+/+^ (WT), *Npc1*^*+/nih*^ (HET) and *Npc1*^*nih/nih*^ (KO) mice at the age of 20–23, 40–43, 60–63 and 84–90 days. Data (*n* = 6–12, mean ± S.D.) were analysed using a two-way ANOVA: *** *P* < 0.001 from *Npc1*^+/+^.

*Npc1*^*nih/nih*^ BALB/c mice with a null allele start developing neurological symptoms such as gait ataxia and tremors around P50. These symptoms progress rapidly, leading to the death of the animals between 11 and 12 weeks of age. Since hepatosplenomegaly is an early indicator of NPC, we focused our analysis on liver and spleen. In line with previous reports, we detected symptoms of visceral disease as early as P20. A major increase in liver weight was observed in *Npc1*^*nih/nih*^ mice when compared with *Npc1*^*nih/+*^ and *Npc1*^+/+^ littermates (data not shown). Hepatomegaly was observed at the earliest time point analysed (P20) and intensified with age. Hematoxylin & eosin (H&E) staining of liver sections of these animals showed gradual increase of lipid-laden cells in the centrilobular area (Fig 1A). Concomitantly, at P20 *Npc1*^*nih/nih*^ mice also presented a marked accumulation of total hepatic cholesterol compared to control animals (Fig 1B). The sphingolipid ceramide (Cer) and the GSLs glucosylceramide (GlcCer), lactosylceramide (LacCer) and globotriaosylceramide (Gb3) were also significantly increased in the liver of P20 *Npc1*^*nih/nih*^ mice compared to *Npc1*^*+/nih*^ and *Npc1*^+/+^ age-matched controls (Fig 1B). Overall the accumulation of the different lipids progressed with age, reaching its maximum at end-stage between 84–90 days of age (Fig 1B).

**Fig 2:**
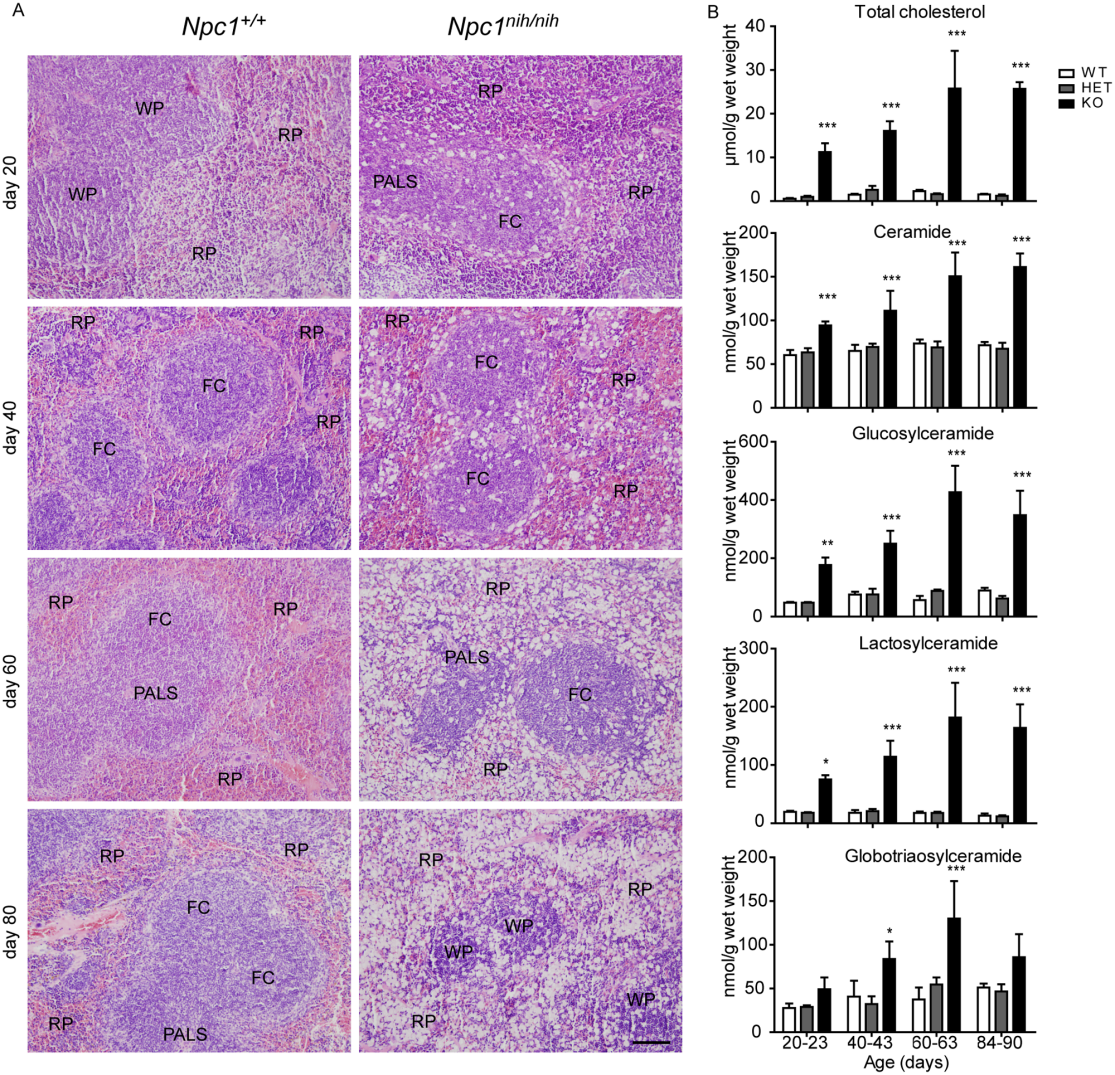
Progressive accumulation of cholesterol and GSLs in *Npc1*^*nih/nih*^ mice spleen. (A) H&E staining of spleen sections of *Npc1*^+/+^ and *Npc1*^*nih/nih*^ at 20, 40, 60 and 80 days of age. FC = follicle centre, PALS = peri-arteriolar lymphocyte sheath, WP = white pulp, RP = red pulp. Scale bar = 100 μm. (B) Levels of total cholesterol, ceramide and GSLs, glucosylceramide, lactosylceramide and globotriaosylceramide in spleen lysates of *Npc1*^+/+^ (WT), *Npc1*^*+/nih*^ (HET) and *Npc1*^*nih/nih*^ (KO) mice at the age of 20–23, 40–43, 60–63 and 84–90 days. Data (*n* = 3–6, mean ± S.D.) were analysed using a two-way ANOVA: * *P* < 0.05, ** *P* < 0.01 and *** *P* < 0.001 from *Npc1*^+/+^.

Interestingly, H&E staining of *Npc1*^*nih/nih*^ mice spleen also revealed a striking increase in lipid-filled cells, which after prolonged disease progression lead to a disruption of splenic architecture (Fig 2A). In the spleen we detected a striking accumulation of several lipid species as well (Fig 2B).

### Gpnmb is expressed in foamy lipid laden cells in liver and spleen of *Npc1*^nih/nih^ mice

**Fig 3:**
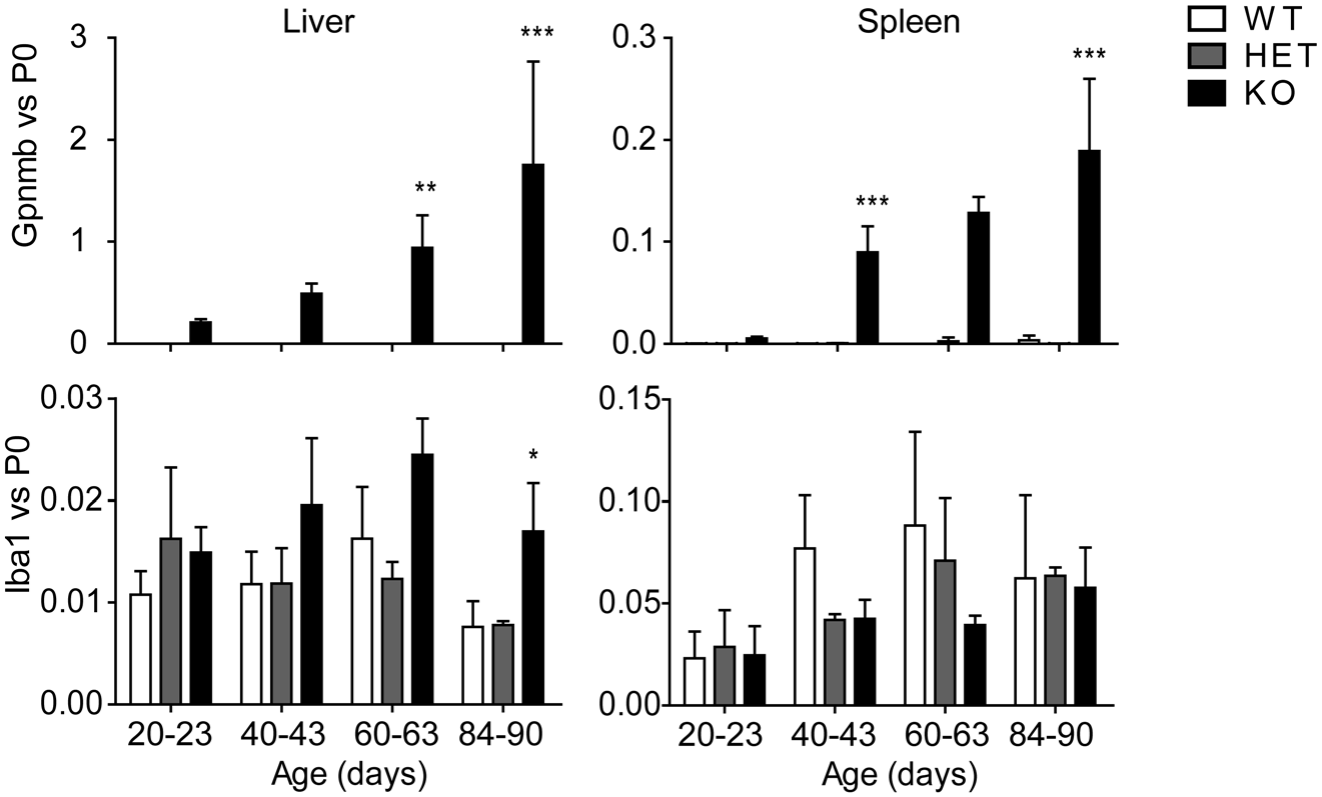
Gpnmb gene expression is increased with disease progression in *Npc1*^*nih/nih*^ mice viscera. Gpnmb (top-panel) and the macrophage marker Iba-1 (bottom-panel) gene expression relative to the house-keeping P0 gene in liver and spleen of *Npc1*^+/+^ (WT), *Npc1*^*+/nih*^ (HET) and *Npc1*^*nih/nih*^ (KO) mice at the age of 20–23, 40–43, 60–63 and 84–90 days. Data (*n* = 3–6, mean ± S.D.) were analysed using a two-way ANOVA: * *P* < 0.05, ** *P* < 0.01 and *** *P* < 0.001 from *Npc1*^+/+^.

In agreement with earlier work [18], we found that Gpnmb mRNA is increased in liver and spleen of *Npc1*^*nih/nih*^ mice (Fig 3). At P20 hepatic Gpnmb expression was already 200-fold higher in *Npc1*^*nih/nih*^ mice compared to age-matched *Npc1*^+/+^ animals and at end-stage this difference was over 2000-fold (Fig 3). In spleen of *Npc1*^*nih/nih*^ mice we also observed a striking increase in Gpnmb expression levels (Fig 3). Cathepsin D expression, a marker earlier found to be elevated in *Npc1* deficient mice [20,37,38], was also increased in liver and spleen of *Npc1*^*nih/nih*^ mice (data not shown).

Gpnmb has been reported to be expressed in macrophages [39]. To establish whether macrophages are indeed the source of elevated Gpnmb in NPC mouse tissues, we analysed the expression levels of the macrophage marker ionized calcium-binding adapter molecule 1 (Iba-1) in liver and spleen of the different animals. In liver of *Npc1*^*nih/nih*^ mice we only observed a significant increase in Iba-1 gene expression compared to controls at 84–90 days of age (Fig 3). In spleen we did not observe increased Iba-1 mRNA expression levels (Fig 3). Expression levels of macrophage marker F4/80 were also analysed and presented a similar pattern as Iba-1 (data not shown).

**Fig 4:**
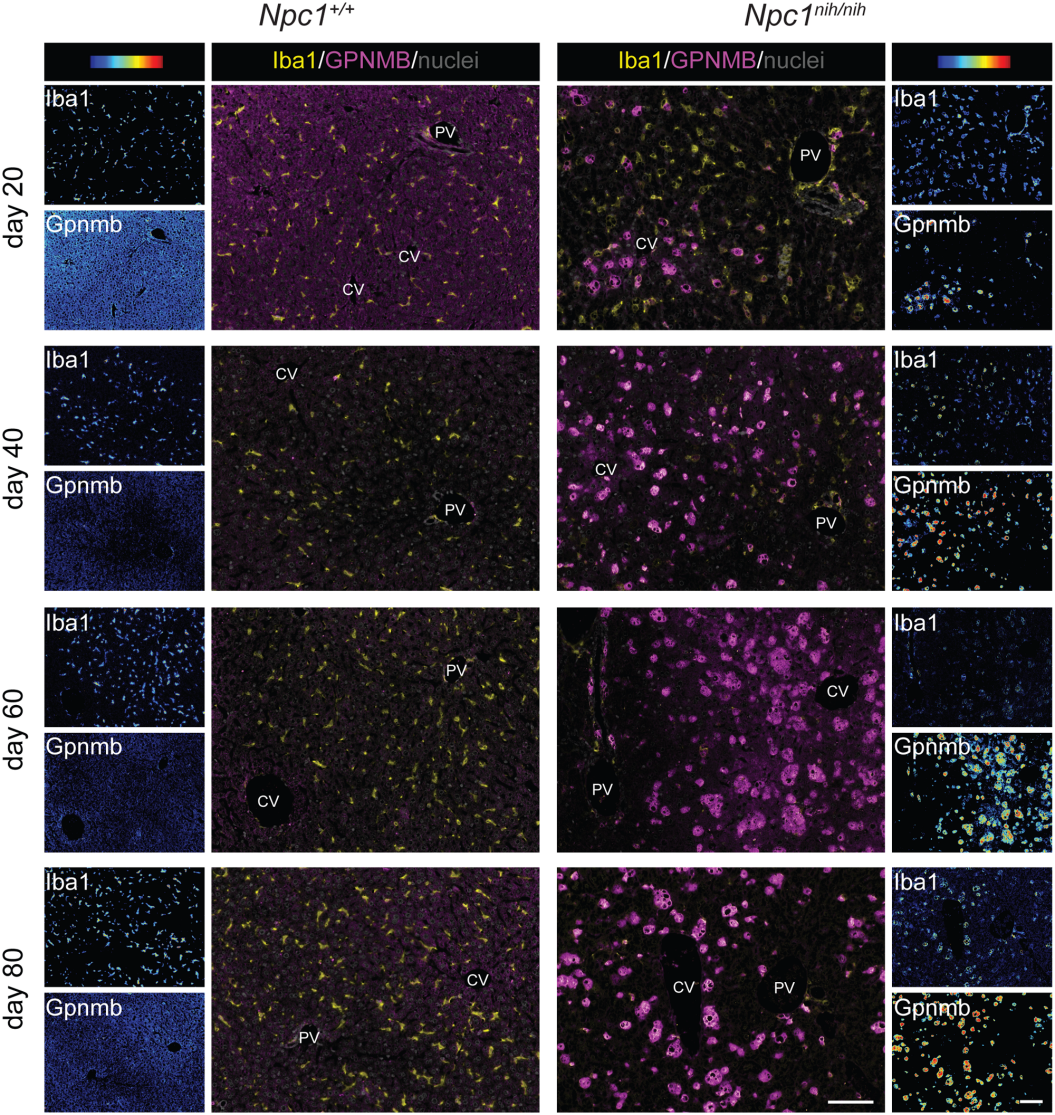
Gpnmb positive cells are increased with disease progression in *Npc1*^*nih/nih*^ mice liver. "Composite" panels show immunostaining of Gpnmb in magenta and Iba-1 in yellow and methylgreen nuclear counterstain of liver of *Npc1*^+/+^ and *Npc1*^*nih/nih*^ at 20, 40, 60 and 80 days of age. Brightfield scans were analysed using spectral imaging, Iba1 and Gpnmb are displayed with a color-coded intensity scale. CV = central vein, PV = portal vein. Scale bar = 100 μm.

**Fig 5:**
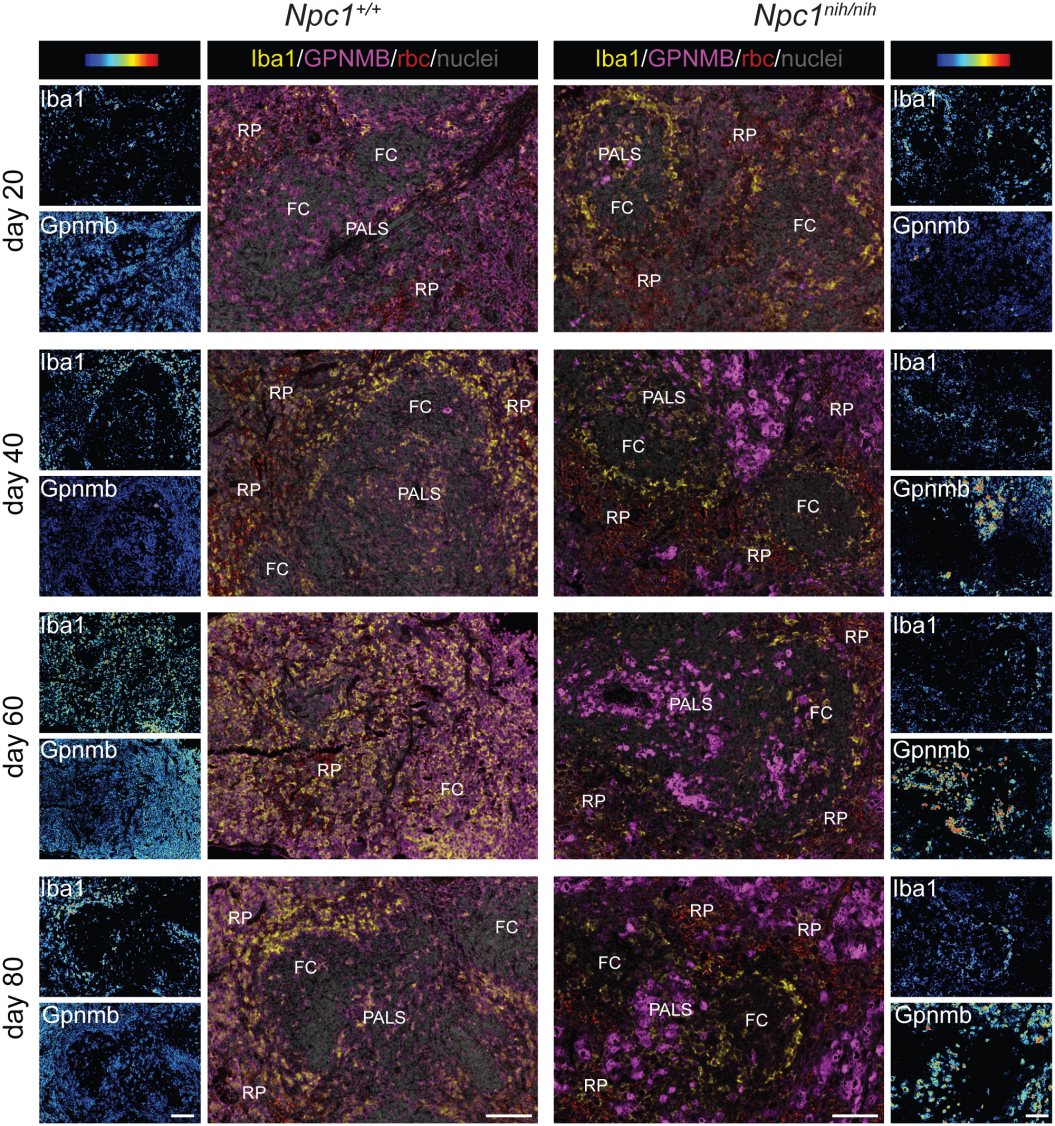
Gpnmb positive cells are increased with disease progression in *Npc1*^*nih/nih*^ mice spleen. "Composite" panels show immunostaining of Gpnmb in magenta and Iba-1 in yellow and methylgreen nuclear counterstain of spleen of *Npc1*^+/+^ and *Npc1*^*nih/nih*^ at 20, 40, 60 and 80 days of age. Brightfield scans were analysed using spectral imaging, Iba1 and Gpnmb are displayed with a color-coded intensity scale. Red blood cells (rbc) were imaged as well (red colour, “composite” panels). FC = follicle centre, PALS = peri-arteriolar lymphocyte sheath, RP = red pulp. Scale bar = 100 μm.

To address the question whether Gpnmb is expressed by macrophages we performed immunohistochemical double staining analysis for Gpnmb and Iba-1 in liver and spleen. Gpnmb protein containing cells strongly increased in numbers with age in liver (Fig 4) and spleen (Fig 5) of *Npc1*^*nih/nih*^ mice. Gpnmb staining abundance per cell also increased with age. All Gpnmb protein containing cells stained positive for Iba1 and appeared swollen, and are therefore most likely lipid-laden macrophages. In liver at day 20, only a minority of Iba1-positive macrophages also stained positive for Gpnmb; these Iba1^pos^Gpnmb^pos^ macrophages are located in acinar zone 3, near the central vein, and contain lipids (Fig 4). The majority of Iba1^pos^Gpnmb^neg^ macrophages are located in the more central acinar zone 2; these cells are increased in size in comparison with Iba1^pos^Gpnmb^neg^ macrophages in *Npc1*^*+/+*^ liver, but do not seem to have accumulated lipids. With age, the number of Iba1^pos^Gpnmb^pos^ lipid-laden macrophages strongly increased in *Npc1*^*nih/nih*^ liver (Fig 4), in full agreement with gene expression data.

In *Npc1*^*nih/nih*^ spleen at day 20 (Fig 5), low numbers of Iba^pos^Gpnmb^pos^ macrophages were detected in the T-lymphocyte rich peri-arteriolar lymphocyte sheath area (PALS). At that stage, lipid-containing Iba1^pos^ macrophages were mostly present in the marginal zone surrounding the white pulp, which encompasses the periarteriolar lymphocyte sheath and the follicles (Fig 2A), but these cells do not express Gpnmb (Fig 5). Lipid-laden Iba1^pos^Gpnmb^pos^ macrophages increasingly populated the red pulp area at days 40, 60 and 80, finally leading to disruption of the splenic architecture (Fig 2A). Remarkable, lipid-laden Iba1^pos^ macrophages in the marginal zone remained Gpnmb negative at all stages examined. This Iba1^pos^Gpnmb^neg^ subset of macrophages also does not express F4/80, whereas F4/80 was detected by single immunostain on adjacent sections in the PALS, follicle centres and red pulp area that contain Iba1^pos^Gpnmb^pos^ lipid-laden macrophages in *Npc1*^*nih/nih*^ mice (not shown).

### RAW264.7 macrophages exposed to the U18666A compound mimic cholesterol-sphingolipid accumulation and Gpnmb upregulation

Considering our finding that macrophages are the main Gpnmb-producing cell type in NPC mice viscera, we performed *in vitro* experiments using the murine macrophage RAW264.7 cell line. The NPC phenotype was induced in these cells using U18666A, an amphipathic steroid drug which is widely used to block intracellular cholesterol trafficking by hindering the exit of free cholesterol from the LE/L compartment [40,41].

**Fig 6:**
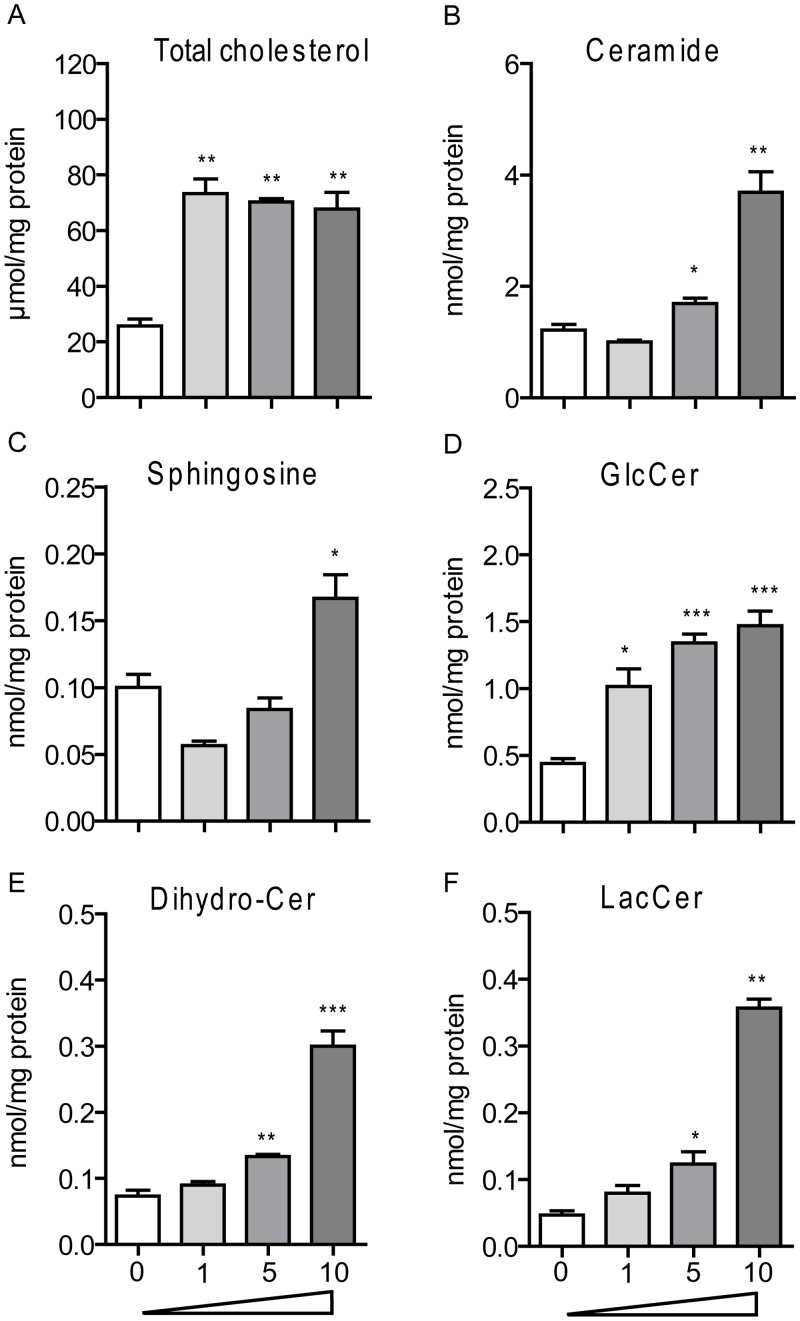
Total Cholesterol and GSLs accumulate in macrophages exposed to U18666A. (A) total cholesterol, (B) ceramide, (C) sphingosine, (D) glucosylceramide (GlcCer), (E) dihydro-ceramide (Dihydro-Cer), and (F) lactosylceramide (LacCer) levels in RAW264.7 murine macrophages exposed to 1, 5 and 10 μM of U18666A for 24 h. Data (*n* = 3, mean ± S.E.M.) were analysed using an unpaired t-test. * *P* < 0.05, ** *P* < 0.01 and *** *P* < 0.001 from DMSO control.

Following 24 hours exposure of RAW264.7 macrophages to various concentrations (1–10 μM) of U18666A, total cholesterol levels were already induced to maximal level with the lowest dose of drug (Fig 6A). At the highest dose sphingosine, ceramide and dihydro-ceramide were also increased (Fig 6B, 6C and 6E). Complex GSLs showed a U18666A dose-dependent increase (Fig 6D and 6F). Thus, the U18666A cell model nicely mimics the lipid accumulation observed in the viscera of *Npc1*^*nih/nih*^ mice.

**Fig 7:**
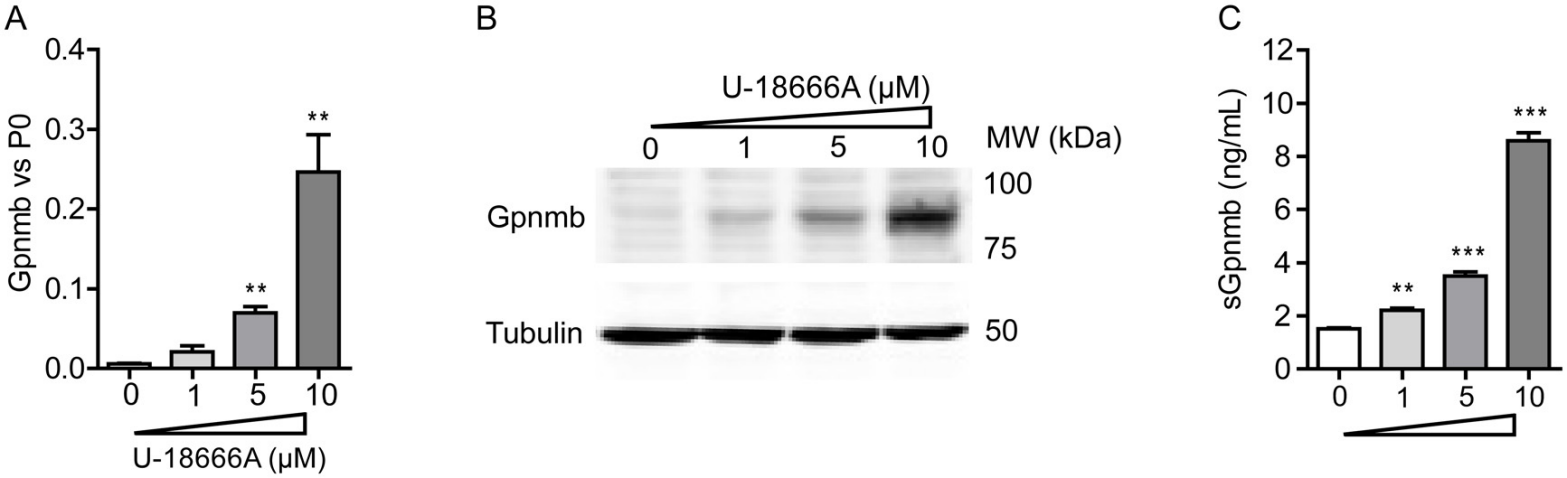
Gpnmb gene and protein expression are increased in macrophages exposed to U18666A. RAW264.7 murine macrophages were exposed to 1, 5 and 10 μM of U18666A for 24 h. (A) Gpnmb mRNA levels and (B) Gpnmb protein levels. (C) Soluble Gpnmb assayed by ELISA of the cell culture medium. Data (*n* = 3 mean ± S.E.M.) were analysed using an unpaired t-test. * *P* < 0.05, ** *P* < 0.01 and *** *P* < 0.001 from DMSO control.

Next, we investigated whether Gpnmb is induced by U18666A. Upon 24 h incubation of RAW264.7 cells with different concentrations of U18666A, Gpnmb was induced dose-dependently both transcriptionally and at the level of protein (Fig 7A and 7B). To explore if Gpnmb is shedded by RAW264.7 cells upon treatment with U18666A, an ELISA assay was used. Soluble Gpnmb (sGpnmb) was found to be increased in a dose-dependent manner in medium of RAW264.7 cells as well (Fig 7C).

### Plasma Gpnmb levels are elevated in *Npc1*^nih/nih^ mice and in NPC patients

**Fig 8:**
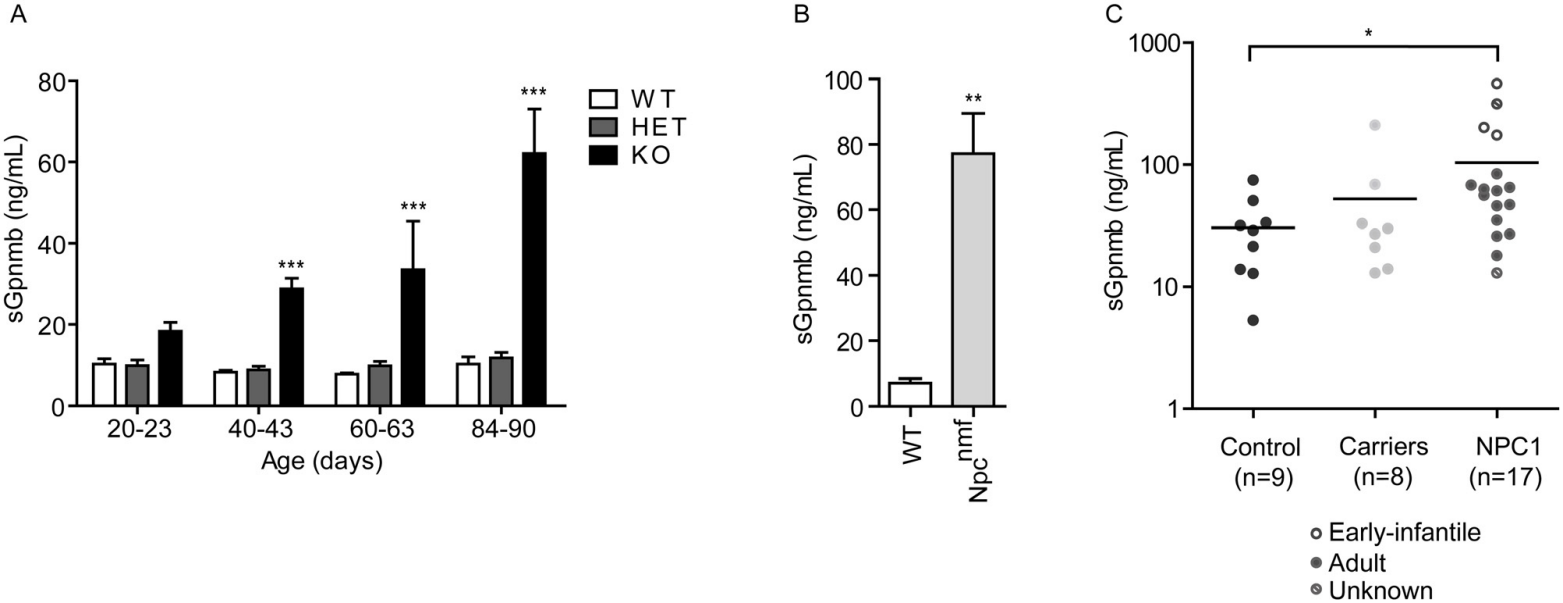
Soluble Gpnmb levels are increased in NPC mice and patient plasma. (A) Plasma sGpnmb levels were assayed by ELISA in plasma of *Npc1*^+/+^ (WT), *Npc1*^*+/nih*^ (HET) and *Npc1*^*nih/nih*^ (KO) mice at the age of 20–23, 40–43, 60–63 and 84–90 days. Data (*n* = 3–6, mean ± S.E.M.) were analysed using a two-way ANOVA: * *P* < 0.05, ** *P* < 0.01 and *** *P* < 0.001 from *Npc1*^+/+^. (B) Plasma levels of sGpnmb in *Npc1*^+/+^ (WT) and *Npc1*^*nmf164*^ mice at 110 days of age. Data (*n* = 3–5, mean ± S.E.M.) were analysed using an unpaired t-test: ** *P* < 0.01. (C) Plasma levels of sGpnmb in healthy volunteers (*n* = 9), carriers of one known mutation on the *Npc1* gene (*n* = 8) and confirmed NPC patients (*n* = 17) detected by ELISA, were analysed using the Matt-Whitney u test: * *P* < 0.05.

Next, we investigated if sGpnmb is elevated in plasma of *Npc1*^*nih/nih*^ mice. Interestingly, we observed an elevation of sGpnmb in plasma of the *Npc1*^*nih/nih*^ animals, which increased over time with disease progression (Fig 8A). At end-stage *Npc1*^*nih/nih*^ mice displayed a 6-fold increase in plasma sGpnmb levels compared with *Npc1*^*+/+*^ or *Npc1*^*nih/+*^ controls. Additionally, we analysed sGpnmb levels in the plasma of the late-onset *Npc1*^nmf164^ mouse model. This model has a point mutation in the *Npc1* gene that corresponds to a single amino acid change (D1005G) in the NPC1 protein and is more representative of the juvenile/classic form of human disease [29]. In plasma of end-stage (110 day-old) *Npc1*^nmf164^ mice the levels of sGpnmb were 8-fold elevated compared with age-matched controls (Fig 8B). Next we analysed if the mouse findings could be translated to the human disease by determination of sGpnmb in plasma of patients diagnosed with NPC not receiving Zavesca treatment. Most patients analysed suffered from the adult form of the disease (n = 12) and three suffered from the early-infantile form (for patient information see S1 Table). Importantly, sGpnmb was found to be significantly elevated in NPC plasma compared to healthy control individuals (Fig 8C). Plasma of NPC carriers (one confirmed mutation) showed a trend of elevated sGpnmb (Fig 8C). Ages between the groups analysed did not differ significantly: NPC (*n* = 17, age 41.3 ± 5.6 [mean ± S.E.M.]), healthy control individuals (*n* = 9, age 42.4 ± 2.8) and NPC carriers (*n* = 8, age 47.2 ± 4.2).

### Gpnmb induction in ‘NPC’ RAW264.7 is glycosphingolipid dependent

**Fig 9:**
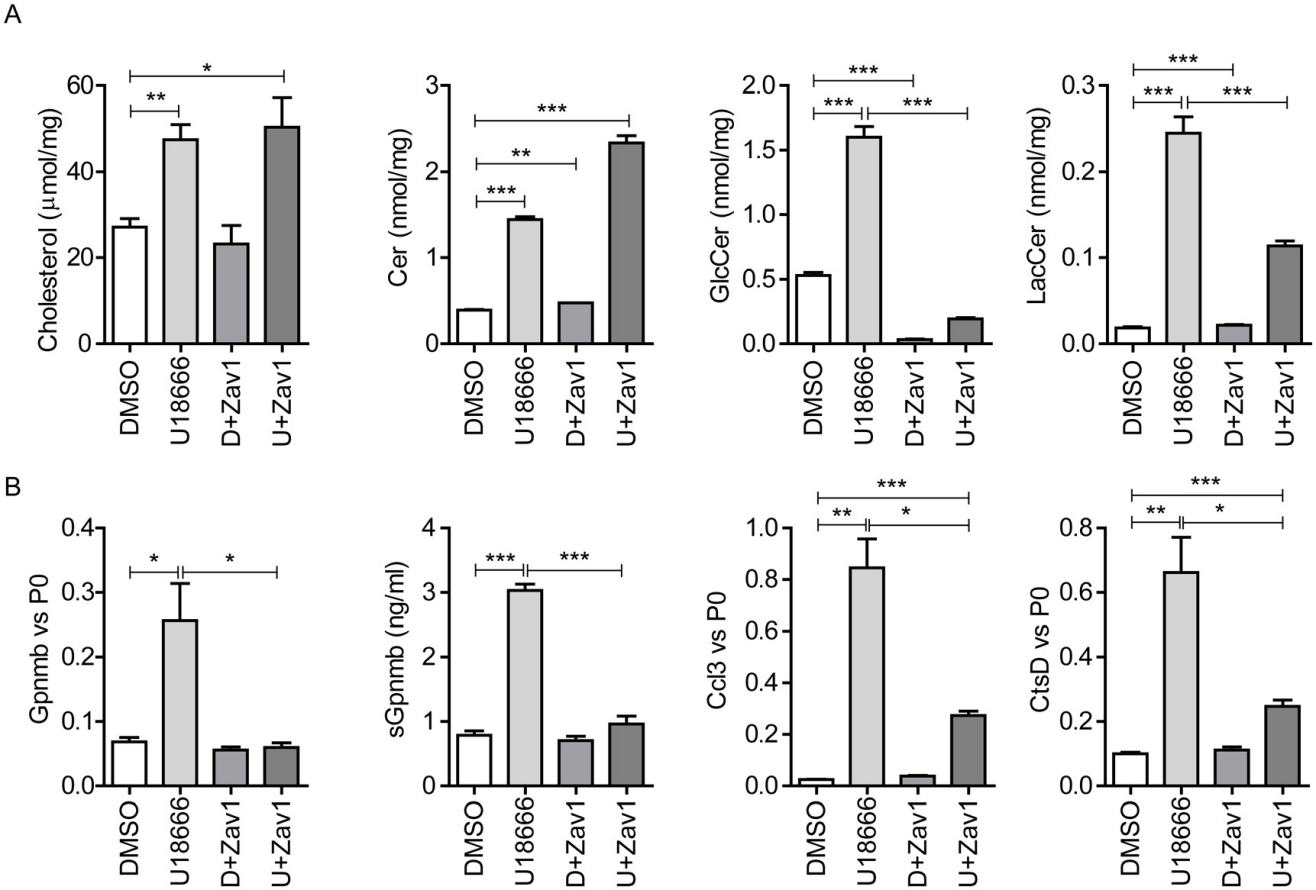
GSL synthesis inhibition abrogates Gpnmb induction. (A) Total cholesterol, ceramide, glucosylceramide (GlcCer) and lactosylceramide (LacCer) levels of RAW264.7 murine macrophages exposed to 10 μM U18666A and/or 250 μM NB-DNJ for 20 h. (B) Gpnmb, Ccl3 and Cathepsin D (CtsD) gene expression, and sGpnmb levels of RAW264.7 cells exposed to 10 μM U18666A and/or 250 μM NB-DNJ for 20 h. Data (*n* = 3 mean ± S.E.M.) were analysed using an unpaired t-test. * *P* < 0.05, ** *P* < 0.01 and *** *P* < 0.001.

In *Npc1*^*nih/nih*^ mice at P20 and RAW264.7 cells exposed to U18666A at the lowest concentration, cholesterol levels were already maximally elevated. Interestingly, build-up of GSLs also occurred and Gpnmb levels seemed to follow the accumulation of GSLs. Therefore we hypothesized that Gpnmb induction might be caused by lysosomal GSLs rather than cholesterol. To this end, we used RAW264.7 cells first treated with U18666A for 8 h followed by an additional 12 h of glucosylceramide synthase (GCS) inhibition with N-butyl-1-deoxynojirimycin (NB-DNJ, Zavesca, 250 μM) to lower GSL levels [42]. At this concentration NB-DNJ not only inhibits GCS, but also partially inhibits GBA and fully GBA2, both responsible for the degradation of GlcCer [43,44]. Cells treated with U18666A, in combination with NB-DNJ or not, presented comparable accumulation of total cholesterol (Fig 9A). Ceramide levels were increased 1.5 fold with the combination treatment (Fig 9A). In contrast, both GlcCer and LacCer were reduced upon NB-DNJ treatment (Fig 9A). Of note, reducing GlcCer levels also deprives GBA and GBA2 of substrate for degradation thus adding to the direct enzyme inhibition by NB-DNJ. Remarkably, Gpnmb gene expression and its secretion were abrogated in the presence of NB-DNJ, suggesting that GSL (GlcCer and LacCer) accumulation, but not cholesterol itself, is triggering Gpnmb induction (Fig 9B). Additionally, other markers for lysosomal stress such as cathepsin D (CtsD) and Ccl3 also showed a marked reduction upon NB-DNJ treatment (Fig 9B).

**Fig 10:**
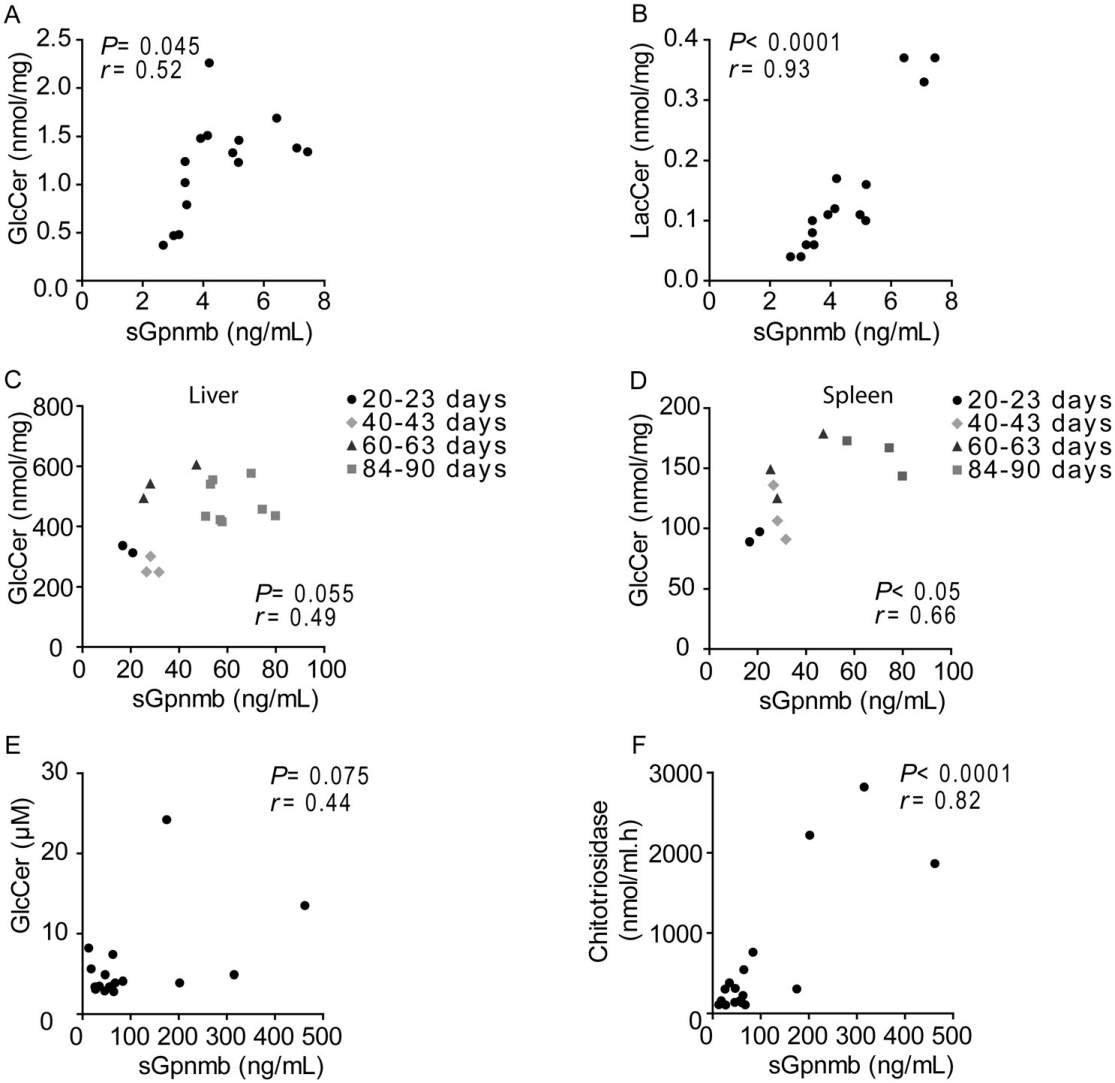
Correlation between glycosphingolipids and sGpnmb levels. Correlation between cell lysate (A) glucosylceramide (GlcCer) or (B) lactosylceramide (LacCer) and sGpnmb from the medium of RAW264.7 cells exposed to 1.25, 2.5, 5 and 10 μM of U18666A for 20 h. Correlation between tissue lysate (C) hepatic or (D) splenic GlcCer levels and plasma sGpnmb of *Npc1*^*nih/nih*^ mice at the age of 20–23, 40–43, 60–63 and 84–90 days. Correlation between plasma (E) GlcCer or (F) chitotriosidase activity and sGpnmb levels from diagnosed NPC patients (*n* = 17). Pearson correlational analysis was used to evaluate correlation.

Next, the relation between sGpnmb and GSL accumulation in the cells was analysed, by exposing RAW264.7 macrophages to different concentrations of U18666A for 20 hours. A dose-dependent increase of GlcCer and LacCer was observed. Interestingly, the GSL levels in the cells significantly correlated with sGpnmb present in the medium (GlcCer: *r* = 0.52, *P* = 0.045, Fig 10A) (LacCer: *r* = 0.93, *P* < 0.0001, Fig 10B). Liver and spleen macrophages of *Npc1*^*nih/nih*^ mice produce Gpnmb that possibly contributes to the pool of sGpnmb in plasma. Therefore, we correlated splenic and hepatic GSLs with plasma sGpnmb levels. We found a positive association between GlcCer levels and sGpnmb, which was significant in the spleen (*r* = 0.66, *P* < 0.05, Fig 10C) and nearly significant in the liver (*r* = 0.49, *P* = 0.055, Fig 10D). As we did not have access to human visceral tissue samples, we then determined if sGpnmb plasma levels were related to GSLs in circulation in NPC patients. The correlation between plasma GlcCer and sGpnmb levels almost reached significance in the patient samples analysed (*r* = 0.44; *P* = 0.075, Fig 10E). Importantly, the levels of sGpnmb in circulation significantly correlated with chitotriosidase enzymatic activity (Fig 10F), a biomarker used to evaluate disease severity and progression in Gaucher disease patients with a deficiency in lysosomal glucocerebrosidase [36] and also used in NPC diagnosis [45].

## Discussion

Reliable biomarkers are essential to monitor disease onset, progression and therapy efficacy. Here we report on the possible use of plasma levels of Gpnmb as marker for the lysosomal storage disorder NPC. We found Gpnmb to be elevated, both in tissue and plasma of *Npc1*^*nih/nih*^ mice, increasing with age and disease progression. The observed induction in tissue Gpnmb gene expression is in line with previous reports [18]. Gpnmb has been found to be highly induced in *Npc1*^*nih/nih*^ mouse tissue arrays and upon bio-statistical analysis connected to innate immune response [18,20]. However, Gpnmb was not ranked as a potential disease plasma correlate [18]. Our detailed immunohistochemical analysis of liver and spleen in *Npc1*^*nih/nih*^ mice, points towards lipid accumulating macrophages as source of sGpnmb, which was further supported by *in vitro* studies using RAW264.7 cells treated with U18666A to mimic the NPC phenotype. The driver of Gpnmb expression in RAW264.7 cells is most likely a GSL, as incubation with the GCS inhibitor N-butyl-1-deoxynojirimycin (NB-DNJ, Zavesca), the only EMA-approved drug for the treatment of NPC, lowered GSLs such as GlcCer and LacCer and concomitantly Gpnmb, while not lowering cholesterol levels.

Additional evidence for a possible role of increased lysosomal lipid pressure in driving Gpnmb expression stems from the observation of Gpnmb-positive cells in the immunohistochemical analysis. In liver of *Npc1*^*nih/nih*^ mice, the number of Gpnmb-positive lipid-laden macrophages strongly increased with age. In the spleen, the macrophages undergoing the highest lipid pressure, the red pulp macrophages, were the predominant Gpnmb-positive cell populations. Red pulp macrophage Gpnmb staining has also been previously observed by others [46]. At this site dysfunctional erythrocytes are cleared from the circulation, thus provoking a high rate of intracellular lipid catabolism. In line with histological observations, we found a positive correlation between plasma sGpnmb and splenic/hepatic GSLs levels, suggesting that the macrophages in these organs substantially contribute to the secreted Gpnmb levels. Additionally, we found sGpnmb to be elevated in a mouse model of the more common juvenile/classic form of the disease, the *Npc1*^*nmf164*^ model.

Importantly, in human subjects suffering from NPC disease sGpnmb was also found to be elevated. This needs to be interpreted with caution as the patient group numbers are limited. GSL levels almost significantly correlated with sGpnmb in patient plasma. Of note, the sGpnmb values correlated nicely with chitotriosidase a well-known macrophage derived LSD marker, which has also been found to be elevated in NPC patients [45]. Correlation of sGpnmb was also observed with CCL18, a chemokine reported to be elevated in GD [47], and oxysterols, cholesterol oxidation products and established specific biomarkers for NPC [17] (S1 Table).

The presence of a soluble form of Gpnmb in NPC is not surprising as several *in vitro* studies already pointed towards the existence of shedded variants of Gpnmb. This was for instance reported in C2C12 myoblasts, melanocytic cells, astrocytes and breast cancer cells [25–28]. Furthermore, in a mouse model of GD, a LSD characterized by a deficient lysosomal acid β-glucosidase (GBA) enzyme causing accumulation of GlcCer levels and derivatives, spleen and hepatic Gpnmb gene expression levels were also found to be elevated [48]. Gpnmb was speculated to serve as potential biomarker [48] and recently elevated levels of Gpnmb have been demonstrated in cerebrospinal fluid (CSF) of a GD mouse model and in CSF of neuropathic GD patients [49]. Of interest we have also found sGpnmb levels to be massively elevated in Gaucher patient plasma (to be published by van Eijk *et al*.). sGpnmb is therefore not a disease specific marker but rather appears to reflect the GSL load, in particular GlcCer, in macrophages. In this context it is important to note that lysosomal GlcCer accumulation has been shown to increase lysosomal pH [50].

Another condition associated with lysosomal alterations is obesity [51]. We have recently shown that Gpnmb is elevated in white adipose tissue macrophages of obese mice and obese subjects [35]. Cholesterol-induced lysosomal stress as observed in LDLR^-/-^ mice fed a western type diet revealed elevated hepatic Gpnmb levels (300-fold) and this was reversed upon GCS inhibition [52]. Taken all data together we propose that cholesterol accumulation is accompanied by GSL build-up, which in turn perturbs lysosomal function in *Npc1*-deficient macrophages driving Gpnmb induction. Our data indicate that macrophages from the liver and spleen are a considerable source of the sGpnmb found in plasma. Nonetheless, we cannot exclude a contribution of neurologically derived sGpnmb, since astrocytes have been shown to secrete Gpnmb [28] and sGpnmb can be detected in the CSF of GD patients [49]. In that regard, we found Gpnmb expression to also be elevated in the cerebellum of *Npc1*-deficient mice compared to wt, being mostly expressed by Iba1-positive microglial cells (S1 Fig).

Our study demonstrates that sGpnmb is a potential plasma marker to follow disease progression in NPC mice. Of note, we show that sGpnmb responds to NB-DNJ treatment *in vitro*, making it a potential candidate for assessing substrate reduction therapy efficacy. The significant elevation of sGpnmb in human NPC is promising and warrants additional analysis in a larger cohort to establish its role as a potential new marker.

## Supporting Information

S1 Fig Gpnmb is increased in the cerebellum of *Npc1*^*nih/nih*^ mice. Sagittal cerebellar sections of 85-days-old wt and *Npc1*^*nih/nih*^ mice immunostained with antibodies against Gpnmb (blue) and Iba-1 (red). Scale bar = 1 mm (top panel), 200 μm (middle panel) and 50 μm (bottom panel).
(TIF)

S1 Table Clinical information of NPC patients. The status of affected or carrier of NPC disease was determined after the exomic sequencing of NPC1 and NPC2 genes, according to the presence of two or one mutations, respectively. Filipin stainings of fibroblasts were conducted to complete the diagnosis. Samples in red are NPC patients and samples in light red are patients with uncertain diagnosis.
(XLS)

S1 Text Supplemental Methods. (DOC)

## References

[1] Vanier MT. Niemann-Pick disease type C. Orphanet J Rare Dis. 2010;5: 1–18. 10.1186/1750-1172-5-16 20525256 PMC2902432

[2] Carstea ED, Morris JA, Coleman KG, Loftus SK, Zhang D, Cummings C, et al Niemann-Pick C1 disease gene: homology to mediators of cholesterol homeostasis. Science. 1997;277: 228–31. 9211849 10.1126/science.277.5323.228

[3] Pentchev PG. Niemann-Pick C research from mouse to gene. Biochim Biophys Acta. 2004;1685: 3–7. 10.1016/j.bbalip.2004.08.005 15465420

[4] Vanier MT. Biochemical studies in Niemann-Pick disease. I. Major sphingolipids of liver and spleen. Biochim Biophys Acta. 1983;750: 178–84. 6824712 10.1016/0005-2760(83)90218-7

[5] Besley GT, Moss SE. Studies on sphingomyelinase and beta-glucosidase activities in Niemann-Pick disease variants. Phosphodiesterase activities measured with natural and artificial substrates. Biochim Biophys Acta. 1983;752: 54–64. 6303436 10.1016/0005-2760(83)90232-1

[6] Salvioli R, Scarpa S, Ciaffoni F, Tatti M, Ramoni C, Vanier MT, et al Glucosylceramidase mass and subcellular localization are modulated by cholesterol in Niemann-Pick disease type C. J Biol Chem. 2004;279: 17674–80. 10.1074/jbc.M313517200 14757764

[7] Patterson MC, Vecchio D, Prady H, Abel L, Wraith JE. Miglustat for treatment of Niemann-Pick C disease: a randomised controlled study. Lancet Neurol. 2007;6: 765–72. 10.1016/S1474-4422(07)70194-1 17689147

[8] Loftus SK, Morris JA, Carstea ED, Gu JZ, Cummings C, Brown A, et al Murine model of Niemann-Pick C disease: mutation in a cholesterol homeostasis gene. Science. 1997;277: 232–5. 9211850 10.1126/science.277.5323.232

[9] Morris MD, Bhuvaneswaran C, Shio H, Fowler S. Lysosome lipid storage disorder in NCTR-BALB/c mice. I. Description of the disease and genetics. Am J Pathol. 1982;108: 140–9. 6765731 PMC1916074

[10] Vázquez MC, del Pozo T, Robledo FA, Carrasco G, Pavez L, Olivares F, et al Alteration of gene expression profile in Niemann-Pick type C mice correlates with tissue damage and oxidative stress. PLoS One. 2011;6: e28777 10.1371/journal.pone.0028777 22216111 PMC3245218

[11] Lopez ME, Klein AD, Hong J, Dimbil UJ, Scott MP. Neuronal and epithelial cell rescue resolves chronic systemic inflammation in the lipid storage disorder Niemann-Pick C. Hum Mol Genet. 2012;21: 2946–60. 10.1093/hmg/dds126 22493001 PMC3373242

[12] Rimkunas VM, Graham MJ, Crooke RM, Liscum L. TNF-{alpha} plays a role in hepatocyte apoptosis in Niemann-Pick type C liver disease. J Lipid Res. 2009;50: 327–33. 10.1194/jlr.M800415-JLR200 18815434 PMC2636917

[13] Sayre NL, Rimkunas VM, Graham MJ, Crooke RM, Liscum L. Recovery from liver disease in a Niemann-Pick type C mouse model. J Lipid Res. 2010;51: 2372–83. 10.1194/jlr.M007211 20418540 PMC2903820

[14] Smith D, Wallom K-L, Williams IM, Jeyakumar M, Platt FM. Beneficial effects of anti-inflammatory therapy in a mouse model of Niemann-Pick disease type C1. Neurobiol Dis. 2009;36: 242–51. 10.1016/j.nbd.2009.07.010 19632328

[15] Maxfield FR, Tabas I. Role of cholesterol and lipid organization in disease. Nature. 2005;438: 612–21. 10.1038/nature04399 16319881

[16] Sagiv Y, Hudspeth K, Mattner J, Schrantz N, Stern RK, Zhou D, et al Cutting edge: impaired glycosphingolipid trafficking and NKT cell development in mice lacking Niemann-Pick type C1 protein. J Immunol. 2006;177: 26–30. Available: http://www.ncbi.nlm.nih.gov/pubmed/16785493 16785493 10.4049/jimmunol.177.1.26

[17] Porter FD, Scherrer DE, Lanier MH, Langmade SJ, Molugu V, Gale SE, et al Cholesterol oxidation products are sensitive and specific blood-based biomarkers for Niemann-Pick C1 disease. Sci Transl Med. 2010;2: 56ra81 10.1126/scitranslmed.3001417 21048217 PMC3170139

[18] Alam MS, Getz M, Safeukui I, Yi S, Tamez P, Shin J, et al Genomic expression analyses reveal lysosomal, innate immunity proteins, as disease correlates in murine models of a lysosomal storage disorder. PLoS One. 2012;7: e48273 10.1371/journal.pone.0048273 23094108 PMC3477142

[19] Alam MS, Getz M, Yi S, Kurkewich J, Safeukui I, Haldar K. Plasma signature of neurological disease in the monogenetic disorder Niemann-Pick Type C. J Biol Chem. 2014;289: 8051–66. 10.1074/jbc.M113.526392 24488491 PMC3961638

[20] Cluzeau CVM, Watkins-Chow DE, Fu R, Borate B, Yanjanin N, Dail MK, et al Microarray expression analysis and identification of serum biomarkers for Niemann-Pick disease, type C1. Hum Mol Genet. 2012;21: 3632–46. 10.1093/hmg/dds193 22619379 PMC3406758

[21] Welford RWD, Garzotti M, Marques Lourenço C, Mengel E, Marquardt T, Reunert J, et al Plasma lysosphingomyelin demonstrates great potential as a diagnostic biomarker for Niemann-Pick disease type C in a retrospective study. PLoS One. 2014;9: e114669 10.1371/journal.pone.0114669 25479233 PMC4257710

[22] Giese A-K, Mascher H, Grittner U, Eichler S, Kramp G, Lukas J, et al A novel, highly sensitive and specific biomarker for Niemann-Pick type C1 disease. Orphanet J Rare Dis. 2015;10: 78 10.1186/s13023-015-0274-1 26082315 PMC4479076

[23] Cologna SM, Jiang X-S, Backlund PS, Cluzeau CVM, Dail MK, Yanjanin NM, et al Quantitative proteomic analysis of niemann-pick disease, type c1 cerebellum identifies protein biomarkers and provides pathological insight. PLoS One. 2012;7: e47845 10.1371/journal.pone.0047845 23144710 PMC3483225

[24] Cologna SM, Cluzeau CVM, Yanjanin NM, Blank PS, Dail MK, Siebel S, et al Human and mouse neuroinflammation markers in Niemann-Pick disease, type C1. J Inherit Metab Dis. 2013; 10.1007/s10545-013-9610-6 PMC3877698 23653225

[25] Rose AAN, Annis MG, Dong Z, Pepin F, Hallett M, Park M, et al ADAM10 releases a soluble form of the GPNMB/Osteoactivin extracellular domain with angiogenic properties. PLoS One. 2010;5: e12093 10.1371/journal.pone.0012093 20711474 PMC2919417

[26] Furochi H, Tamura S, Mameoka M, Yamada C, Ogawa T, Hirasaka K, et al Osteoactivin fragments produced by ectodomain shedding induce MMP-3 expression via ERK pathway in mouse NIH-3T3 fibroblasts. FEBS Lett. 2007;581: 5743–50. 10.1016/j.febslet.2007.11.036 18036345

[27] Hoashi T, Sato S, Yamaguchi Y, Passeron T, Tamaki K, Hearing VJ. Glycoprotein nonmetastatic melanoma protein b, a melanocytic cell marker, is a melanosome-specific and proteolytically released protein. FASEB J. 2010;24: 1616–29. 10.1096/fj.09-151019 20056711 PMC2879953

[28] Tanaka H, Shimazawa M, Kimura M, Takata M, Tsuruma K, Yamada M, et al The potential of GPNMB as novel neuroprotective factor in amyotrophic lateral sclerosis. Sci Rep. 2012;2: 1–11. 10.1038/srep00573 PMC3417778 22891158

[29] Maue RA, Burgess RW, Wang B, Wooley CM, Seburn KL, Vanier MT, et al A novel mouse model of Niemann-Pick type C disease carrying a D1005G-Npc1 mutation comparable to commonly observed human mutations. Hum Mol Genet. 2012;21: 730–50. 10.1093/hmg/ddr505 22048958 PMC3263988

[30] De Boer OJ, van der Meer JJ, Teeling P, van der Loos CM, Idu MM, van Maldegem F, et al Differential expression of interleukin-17 family cytokines in intact and complicated human atherosclerotic plaques. J Pathol. 2010;220: 499–508. 10.1002/path.2667 20020510

[31] Gold H, Mirzaian M, Dekker N, Joao Ferraz M, Lugtenburg J, Codée JDC, et al Quantification of Globotriaosylsphingosine in Plasma and Urine of Fabry Patients by Stable Isotope Ultraperformance Liquid Chromatography-Tandem Mass Spectrometry. Clin Chem. 2012;59: 1–10. 10.1373/clinchem.2012.192138 23237761

[32] Bligh EG, Dyer WJ. A rapid method of total lipid extraction and purification. Can J Biochem Physiol. 1959;37: 911–7. 13671378 10.1139/o59-099

[33] Folch J, Lees M, Sloane Stanley GH. A simple method for the isolation and purification of total lipides from animal tissues. J Biol Chem. 1957;226: 497–509. 13428781

[34] Heider JG, Boyett RL. The picomole determination of free and total cholesterol in cells in culture. J Lipid Res. 1978;19: 514–8. 96199

[35] Gabriel TL, Tol MJ, Ottenhof R, van Roomen C, Aten J, Claessen N, et al Lysosomal Stress in Obese Adipose Tissue Macrophages Contributes to MITF-Dependent Gpnmb Induction. Diabetes. 2014;63: 3310–3323. 10.2337/db13-1720 24789918

[36] Hollak CE, van Weely S, van Oers MH, Aerts JM. Marked elevation of plasma chitotriosidase activity. A novel hallmark of Gaucher disease. J Clin Invest. 1994;93: 1288–92. 10.1172/JCI117084 8132768 PMC294082

[37] German DC, Quintero EM, Liang C, Xie C, Dietschy JM. Degeneration of neurons and glia in the Niemann-Pick C mouse is unrelated to the low-density lipoprotein receptor. Neuroscience. 2001;105: 999–1005. 11530237 10.1016/s0306-4522(01)00230-5

[38] Amritraj A, Wang Y, Revett TJ, Vergote D, Westaway D, Kar S. Role of cathepsin D in U18666A-induced neuronal cell death: potential implication in Niemann Pick Type C disease pathogenesis. J Biol Chem. 2013;288: 3136–52. 10.1074/jbc.M112.412460 23250759 PMC3561536

[39] Ripoll VM, Irvine KM, Ravasi T, Sweet MJ, Hume DA. Gpnmb is induced in macrophages by IFN-gamma and lipopolysaccharide and acts as a feedback regulator of proinflammatory responses. J Immunol. 2007;178: 6557–66. 17475886 10.4049/jimmunol.178.10.6557

[40] Liscum L, Faust JR. The intracellular transport of low density lipoprotein-derived cholesterol is inhibited in Chinese hamster ovary cells cultured with 3-beta-[2-(diethylamino)ethoxy]androst-5-en-17-one. J Biol Chem. 1989;264: 11796–806. 2745416

[41] Härmälä AS, Pörn MI, Mattjus P, Slotte JP. Cholesterol transport from plasma membranes to intracellular membranes is inhibited by 3 beta-[2-(diethylamino)ethoxy]androst-5-en-17-one. Biochim Biophys Acta. 1994;1211: 317–25. 8130265 10.1016/0005-2760(94)90156-2

[42] Platt FM, Neises GR, Karlsson GB, Dwek RA, Butters TD. N-butyldeoxygalactonojirimycin inhibits glycolipid biosynthesis but does not affect N-linked oligosaccharide processing. J Biol Chem. 1994;269: 27108–14. 7929454

[43] Wennekes T, van den Berg RJBHN, Donker W, van der Marel GA, Strijland A, Aerts JMFG, et al Development of adamantan-1-yl-methoxy-functionalized 1-deoxynojirimycin derivatives as selective inhibitors of glucosylceramide metabolism in man. J Org Chem. 2007;72: 1088–97. 10.1021/jo061280p 17243712

[44] Overkleeft HS, Renkema GH, Neele J, Vianello P, Hung IO, Strijland A, et al Generation of specific deoxynojirimycin-type inhibitors of the non-lysosomal glucosylceramidase. J Biol Chem. 1998;273: 26522–7. 9756888 10.1074/jbc.273.41.26522

[45] Ries M, Schaefer E, Lührs T, Mani L, Kuhn J, Vanier MT, et al Critical assessment of chitotriosidase analysis in the rational laboratory diagnosis of children with Gaucher disease and Niemann-Pick disease type A/B and C. J Inherit Metab Dis. 2006;29: 647–52. 10.1007/s10545-006-0363-3 16972172

[46] Li B, Castano AP, Hudson TE, Nowlin BT, Lin S-L, Bonventre JV, et al The melanoma-associated transmembrane glycoprotein Gpnmb controls trafficking of cellular debris for degradation and is essential for tissue repair. FASEB J. 2010;24: 4767–81. 10.1096/fj.10-154757 20709912 PMC2992370

[47] Boot RG, Verhoek M, Langeveld M, Renkema GH, Hollak CEM, Weening JJ, et al CCL18: A urinary marker of Gaucher cell burden in Gaucher patients. J Inherit Metab Dis. 2006;29: 564–571. 10.1007/s10545-006-0318-8 16736095

[48] Mistry PK, Liu J, Yang M, Nottoli T, McGrath J, Jain D, et al Glucocerebrosidase gene-deficient mouse recapitulates Gaucher disease displaying cellular and molecular dysregulation beyond the macrophage. Proc Natl Acad Sci U S A. 2010;107: 19473–8. 10.1073/pnas.1003308107 20962279 PMC2984187

[49] Zigdon H, Savidor A, Levin Y, Meshcheriakova A, Schiffmann R, Futerman AH. Identification of a Biomarker in Cerebrospinal Fluid for Neuronopathic Forms of Gaucher Disease. PLoS One. 2015;10: e0120194 10.1371/journal.pone.0120194 25775479 PMC4361053

[50] Sillence DJ. Glucosylceramide modulates endolysosomal pH in Gaucher disease. Mol Genet Metab. 2013;109: 194–200. 10.1016/j.ymgme.2013.03.015 23628459

[51] Xu X, Grijalva A, Skowronski A, van Eijk M, Serlie MJ, Ferrante AW. Obesity activates a program of lysosomal-dependent lipid metabolism in adipose tissue macrophages independently of classic activation. Cell Metab. 2013;18: 816–30. 10.1016/j.cmet.2013.11.001 24315368 PMC3939841

[52] Lombardo E, van Roomen CPaa, van Puijvelde GH, Ottenhoff R, van Eijk M, Aten J, et al Correction of liver steatosis by a hydrophobic iminosugar modulating glycosphingolipids metabolism. PLoS One. 2012;7: e38520 10.1371/journal.pone.0038520 23056165 PMC3466229

