# Supplementary figures and images for: Gpnmb Is a Potential Marker for the Visceral Pathology in Niemann-Pick Type C Disease
Source: PLoS One. 2016 Jan 15;11(1):e0147208. doi: 10.1371/journal.pone.0147208 (PMC4714856; doi:10.1371/journal.pone.0147208)

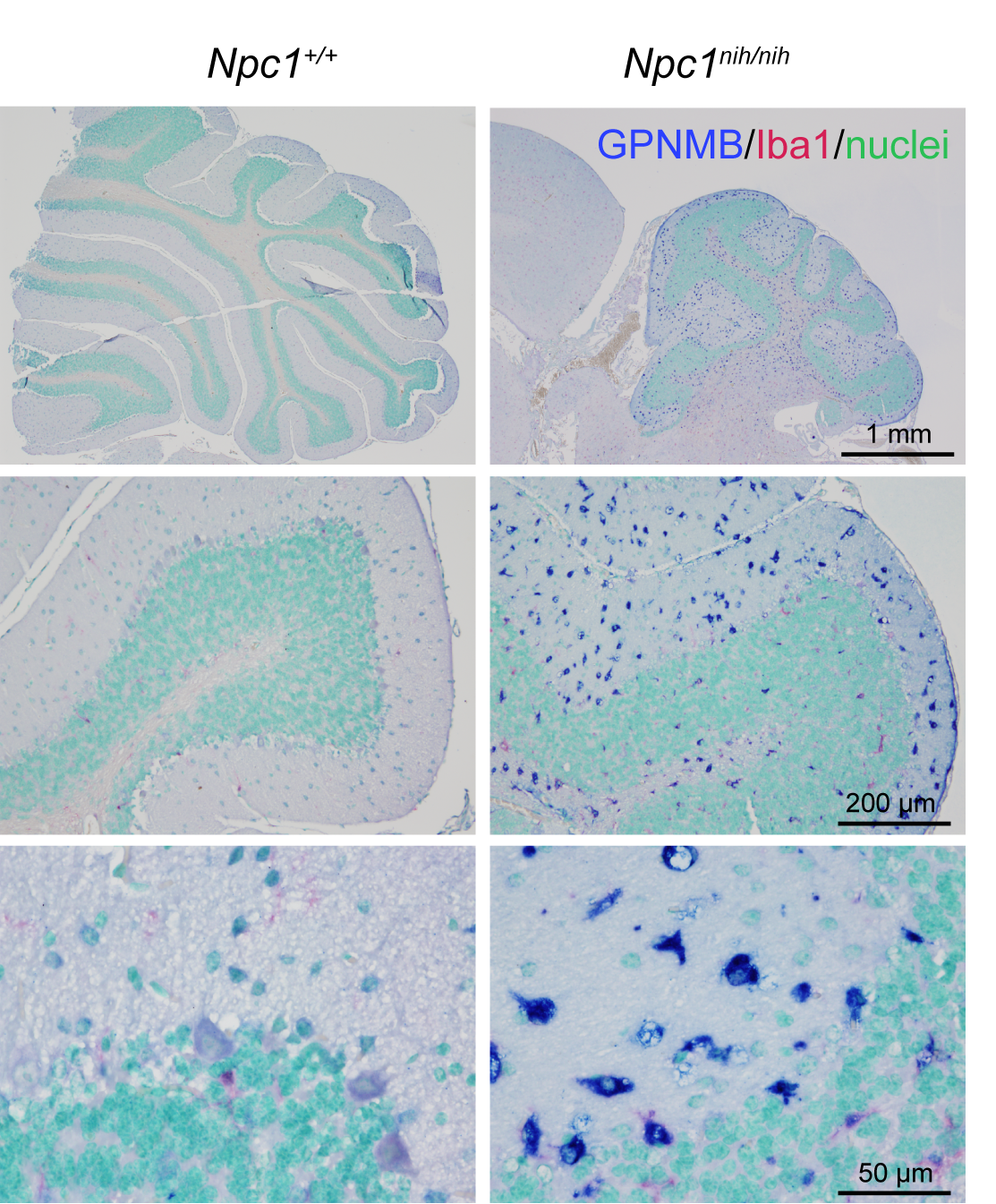

Supplement: S1 Fig — Sagittal cerebellar sections of 85-days-old wt and Npc1nih/nih mice immunostained with antibodies against Gpnmb (blue) and Iba-1 (red). Scale bar = 1 mm (top panel), 200 μm (middle panel) and 50 μm (bottom panel). (TIF) [file pone.0147208.s001.tif]
