# Supplementary material for: Gpnmb Is a Potential Marker for the Visceral Pathology in Niemann-Pick Type C Disease
Source: PLoS One. 2016 Jan 15;11(1):e0147208. doi: 10.1371/journal.pone.0147208 (PMC4714856; doi:10.1371/journal.pone.0147208)
Supplement: S1 Text — (DOC) [file pone.0147208.s003.doc]

**Supplemental Methods**

**Filipin staining and measurement of CCL18 and oxysterols**

Performed as previously described [1,2].

**Immunohistology brain sections for Gpnmb and Iba1**

Sequential double immunostaining was performed on 4-µm-thick sections of formalin-fixed paraffin-embedded tissue. After deparaffination and rehydration, heat-induced epitope retrieval was done in citrate, pH 6, for 10 min at 100 C. Sections were sequentially incubated with polyclonal goat IgG anti-Gpnmb (AF2330; R&D Systems, Abingdon, UK), rabbit IgG anti-goat IgG (6160-01; Southern Biotech, Birmingham, AL, USA), alkaline phosphatase-conjugated goat IgG anti-rabbit IgG (BrightVision; ImmunoLogic, Duiven, The Netherlands), and Vector Blue substrate (Vector Laboratories, Burlingame, CA) in presence of levamisole. Next, heat-induced epitope retrieval was performed again, followed by incubation with rabbit IgG anti-Iba1 (019-19741; Wako Pure Chemical Industries, Osaka, Japan), alkaline phosphatase-conjugated goat IgG anti-rabbit IgG (BrightVision), and Vector Red substrate in presence of levamisole. Sections were counterstained with methyl green and were mounted using AquaMount. The reverse staining sequence was applied as well, i.e. immunostaining for Iba1 followed by immunostaining for Gpnmb.

**Supplemental References**

1. Cebolla JJ, De Castro-Orós I, Irún P, Alfonso P, López de Frutos L, Andrade-Campos M, et al. Experience with 7-ketocholesterol and ccl18/parc as surrogated biomarkers in a series of Spanish Niemann–Pick disease type C patients. Mol Genet Metab. 2015;114: S29. doi:10.1016/j.ymgme.2014.12.047

2. Boven, LA, Meurs M van, Boot, R, Mehta, A, Boon, L, Aerts, J, et al. Gaucher Cells Demonstrate a Distinct Macrophage Phenotype and Resemble Alternatively Activated Macrophages. Am J Clin Pathol. 2004;122: 359–369. doi:10.1309/BG5V-A8JR-DQH1-M7HN
